# Pathologic complete response and survival after neoadjuvant chemotherapy in cT1-T2/N0 HER2+ breast cancer

**DOI:** 10.1038/s41523-022-00433-x

**Published:** 2022-05-12

**Authors:** Selena J. An, Emilie D. Duchesneau, Paula D. Strassle, Katherine Reeder-Hayes, Kristalyn K. Gallagher, David W. Ollila, Stephanie M. Downs-Canner, Philip M. Spanheimer

**Affiliations:** 1 Department of Surgery, University of North Carolina, Chapel Hill, NC USA; 2 Department of Epidemiology, University of North Carolina, Chapel Hill, NC USA; 3 Division of Intramural Research, National Institute on Minority Health and Health Disparities, National Institutes of Health, Bethesda, MD USA; 4 Lineberger Comprehensive Cancer Center, University of North Carolina, Chapel Hill, NC USA; 5 Department of Medicine, Division of Oncology, University of North Carolina at Chapel Hill, Chapel Hill, NC USA

**Keywords:** Breast cancer, Chemotherapy

## Abstract

Women with small HER2+ breast cancers may have excellent prognosis with adjuvant single-agent chemotherapy and HER2-targeted therapy. The role of de-escalated therapy in the neoadjuvant setting, however, remains uncertain. We conducted a cohort study of adult women with T1-2/cN0 HER2+ breast cancer diagnosed 2013–2016 in the National Cancer Database treated with neoadjuvant chemotherapy (NAC) and HER2-targeted therapy. Factors associated with pathologic complete response (pCR) and overall survival were examined. In total, 6994 patients were included, 32% cT1 and 68% cT2. Multi-agent NAC was given to 90% of women while single-agent NAC was given to 10% of women. pCR was achieved in 46% of cT2 patients and 43% of cT1, and in 46% of patients treated with multi-agent versus 38% single agent. Patients receiving multi-agent chemotherapy were younger, had fewer comorbidities, and had higher cT stage and grade. In all patients, pCR was associated with improved survival (*p* < 0.01). Multi-agent chemotherapy (OR 1.3, *p* = 0.003), hormone receptor negative (OR 2.6, *p* < 0.001), higher grade (OR 2.2, *p* < 0.001), younger age (OR 1.4, *p* = 0.011), and later year of diagnosis (OR 1.3, *p* = 0.005) were associated with achieving pCR. Multi-agent chemotherapy was associated with higher likelihood of pCR, but this effect was modest compared to other factors. Single-agent NAC with HER2-directed therapy in selected patients may provide excellent outcome with reduced toxicity, while allowing escalated therapy in the adjuvant setting for patients with residual disease. Prospective studies are needed to determine effects of de-escalation in the neoadjuvant setting on survival and optimal selection strategies.

## Introduction

Breast cancer is among the most common cancers for women in the U.S. and a leading cause of death^1^. HER2+ breast cancers account for ~14% of cases^2^. HER2-amplification was previously associated with high recurrence rates and poor survival^3^; however, development of targeted therapy in the form of monoclonal antibodies and more recently antibody-drug conjugates to HER2 given in combination with cytotoxic chemotherapy has dramatically improved outcomes^4–7^.

Current standard of care for HER2+ breast cancer includes a combination of chemotherapy with HER2-targeted therapy, surgery, radiotherapy, and endocrine therapy depending on estrogen receptor status^8^. Chemotherapy and HER2-targeted therapy can be delivered in either the neoadjuvant or adjuvant setting, but neoadjuvant chemotherapy (NAC) can be used to increase eligibility for breast-conserving surgery and decrease the extent of axillary surgery^8,9^. In addition, response to neoadjuvant therapy, including pathologic complete response (pCR), strongly predicts recurrence and survival^10–12^. For patients who have residual disease after neoadjuvant therapy, escalation of therapy with adjuvant regimens, including with HER2 antibody conjugated chemotherapy (T-DM1), has been shown to increase disease-free survival, demonstrating the use of NAC to identify patients in need of more aggressive treatment^13,14^.

On the other hand, for patients with node-negative small HER2+ breast cancers <3 cm, the APT trial demonstrated that a regimen of single-agent adjuvant paclitaxel with trastuzumab (TH) confers a very low risk of recurrence with a favorable profile of side effects^15^. Trials of de-escalated neoadjuvant therapy using APT-style taxane regimens are ongoing, but multi-agent chemotherapy regimens including carboplatin or anthracyclines in addition to taxanes remain the current standard of care in neoadjuvant therapy of HER2+ breast cancer. Multi-agent chemotherapy regimens are associated with significant side effects including nausea, vomiting, diarrhea, neuropathy, neutropenia, and cardiotoxicity, which can be worsened with HER2-targeted therapy^15–17^.

For small, node-negative HER2+ breast cancer, the optimal treatment sequence remains unclear. NAC risks overtreatment of patients who potentially could have excellent survival and less toxicity with taxane/trastuzumab regimens in the adjuvant setting, while surgery first risks under-treatment by failing to identify patients with residual disease who could benefit from escalated adjuvant therapy. Currently, the decision for surgery/chemotherapy sequence is made in node-negative patients largely based on clinical T stage, which may not be the optimal stratification metric. We hypothesize that NAC with de-escalated taxane/trastuzumab regimens may be the preferred strategy for patients with T1/T2 N0 HER2+ breast cancer to minimize toxicity and identify patients with residual disease in need of escalated systemic therapy. In this study, we examine outcomes of women with early stage, node-negative HER2+ breast cancer treated with single (NCDB surrogate for taxane/trastuzumab) or multi-agent NAC and the association between treatment, pCR, and survival using a large national database.

## Results

### Patient characteristics

This study included 6994 patients diagnosed between 2013 and 2015 with clinical T1/T2, clinical N0 HER2+ breast cancer treated with NAC, and HER2-targeted therapy followed by surgical resection (Table 1). Of those patients, 2242 (32%) were clinical T1 and 4752 (68%) were clinical T2. Median age was 53 years (IQR 45–62). Median follow-up was 19.4 months (IQR 11.5–28.8). The majority of patients had a Charlson Comorbidity Index of 0 (88%) and were White (83%). The most common histology was ductal (90%) and the majority of patients had tumors that were positive for estrogen (67%) and progesterone (54%) receptors. Most tumors were poorly differentiated or undifferentiated (55%), followed by moderately differentiated (40%).

**Table 1:** Demographics and clinical characteristics of patients with HER2-amplified breast cancer who received NAC and HER2-targeted therapy, stratified by NAC regimen.

		NAC regimen	
	Overall *N* = 6994	Single agent *n* = 662	Multi-agent *n* = 6253
Age (median, IQR)	53 (45–62)	60 (49–70)	53 (44–61)
Age category (no, %)			
<40	954 (13.6)	54 (8.2)	892 (14.3)
40–49	1700 (24.3)	112 (16.9)	1565 (25.0)
50–59	2147 (30.7)	161 (24.3)	1964 (31.4)
60–69	1579 (22.6)	162 (24.5)	1400 (22.4)
70–79	549 (7.8)	133 (20.1)	408 (6.5)
80+	65 (0.9)	40 (6.0)	24 (0.4)
Race (no, %)			
White	5772 (82.9)	562 (85.2)	5145 (82.7)
Black	745 (10.7)	61 (9.2)	678 (10.9)
Other^a^	443 (6.4)	37 (5.6)	399 (6.4)
Charlson Comorbidity Index (no, %)			
0	6150 (87.9)	563 (85.0)	5518 (88.2)
1	685 (9.8)	73 (11.0)	602 (9.6)
≥2	159 (2.3)	26 (3.9)	133 (2.1)
cT stage (no, %)			
cT1	2242 (32.1)	273 (41.2)	1934 (30.9)
cT2	4752 (67.9)	389 (58.8)	4319 (69.1)
Histology (no, %)			
Ductal	6280 (89.8)	589 (89.0)	5620 (89.9)
Lobular	559 (8.0)	56 (8.5)	498 (8.0)
Other	155 (2.2)	17 (2.6)	135 (2.2)
Hormone receptor status (no, %)			
ER+	4709 (67.4)	444 (67.1)	4206 (67.3)
PR+	3802 (54.4)	361 (54.5)	3401 (54.5)
Grade (no, %)			
Well-differentiated	312 (4.8)	40 (6.5)	267 (4.6)
Moderately differentiated	2610 (39.8)	265 (42.9)	2311 (39.4)
Poorly or undifferentiated	3637 (55.5)	313 (50.6)	3290 (56.1)
Type of surgery (no, %)			
Mastectomy	3444 (49.3)	308 (46.5)	3088 (49.4)
Breast-conserving surgery	3547 (50.7)	354 (53.5)	3162 (50.6)
Lymph node surgery (no, %)			
ALND	1755 (25.2)	178 (26.9)	1550 (24.9)
SLNB	4967 (71.2)	441 (66.6)	4479 (71.9)
None	252 (3.6)	43 (6.5)	204 (3.3)
Radiation therapy (no, %)	3924 (56.2)	363 (55.4)	3527 (56.5)
Endocrine therapy (no, %)	4298 (62.7)	396 (61.8)	3855 (62.9)

*NAC* neoadjuvant chemotherapy, *ALND* axillary lymph node dissection, *SLNB* sentinel lymph node biopsy.

^a^Other race categories were collapsed due to small sample sizes. “Other” race includes American Indian, Aleutian, Eskimo, Asian, Pacific Islander, and other races.

Treatment characteristics of the cohort showed 49% of patients undergoing mastectomy and 51% breast-conserving surgery (Table 1). The axilla was evaluated by sentinel lymph node biopsy (SLNB) alone in 71% of patients and axillary lymph node dissection (ALND) with or without SLNB in 25% of patients. Axillary staging was not performed in 4% of patients. All patients received HER2-targeted therapy. Chemotherapy regimen was a single agent in 10% of patients and multi-agent in 90% of patients. Treatment characteristics of patients treated with single-agent and multi-agent chemotherapy are listed in Table 1. Patients treated with multi-agent chemotherapy were younger, had fewer comorbidities, higher T stage, and higher grade than patients treated with single-agent regimens. Of the 2242 cT1 patients, 405 (18%) were T1a/b, 1591 (71%) were T1c, and 246 (11%) were T1 not otherwise specified. T1a/b tumors were more likely to be treated with single-agent chemotherapy (29%) compared to T1c (11%).

### Pathologic response to neoadjuvant chemotherapy

Pathologic staging after NAC, by type of chemotherapy and clinical stage, are presented in Table 2. Pathologic complete response was achieved in a total of 2831 women, 43% of cT1 patients and 46% of cT2 patients. In bivariate analyses, patients who received multi-agent chemotherapy had higher rate of pCR (46% versus 37%) compared to single agent, and hormone receptor-positive patients had a lower rate of pCR compared to hormone receptor-negative (37% versus 63%). pCR by chemotherapy in T1a/b compared to T1c patients is presented in Supplementary Table 1. In a multivariable-adjusted analysis (Table 3), multi-agent chemotherapy (odds ratio [OR] 1.34, 95% CI 1.10–1.62) was associated with higher odds of achieving pCR. Hormone receptor-positive status was associated with lower odds of achieving pCR (OR 0.38, 95% CI 0.34–0.43). Moderately differentiated (versus well-differentiated: OR 1.59, 95% CI 1.20–2.11) and poorly/undifferentiated (versus well-differentiated: OR 2.17, 95% CI 1.64–2.88), and later years of diagnosis (2014: OR 1.19, 95% CI 1.02–1.40; 2015: OR 1.25, 95% CI 1.07–1.45) were also associated with achieving pCR.

**Table 2:** Pathologic staging by NAC regimen and pathologic T stage.

	Single-agent NAC		Multi-agent NAC	
	cT1N0 *n* = 273	cT2N0 *n* = 389	cT1N0 *n* = 1934	cT2N0 *n* = 4319
ypT stage (no, %)^a^				
pT0	80 (29.3)	110 (28.3)	677 (35.0)	1557 (36.1)
pT1	148 (54.2)	33 (8.5)	889 (46.0)	1493 (34.6)
pT2+	11 (4.0)	74 (21.9)	94 (5.7)	595 (16.3)
ypN stage (no, %)^b^				
pN0	228 (83.5)	311 (79.9)	1632 (84.4)	3682 (85.3)
pN+	22 (8.1)	43 (11.1)	170 (8.8)	474 (11.0)
pCR (no, %)^c^	90 (32.9)	133 (38.2)	764 (43.9)	1809 (46.6)

*NAC* neoadjuvant chemotherapy, *pCR* pathologic complete response (pT0 pN0).

^a^Missing data from 161 women with cT1N0 disease and 367 women with cT2N0 disease were excluded from the analysis.

^b^Missing data from 158 women with cT1N0 disease and 299 women with cT2N0 disease were excluded from the analysis.

^c^Missing data from 229 women with cT1N0 disease and 480 women with cT2N0 disease were excluded from the analysis. Breast-only pCR in the single-agent neoadjuvant chemotherapy group was suppressed due to cell size reporting requirements.

**Table 3:** Multivariable logistic regression model of predictors of achieving pCR^a^.

	OR	(95% CI)	*p*-value
cT2 stage (ref = cT1)	1.07	(0.95, 1.20)	0.268
Multi-agent NAC (ref = single agent)	1.34	(1.10, 1.62)	0.003
Hormone receptor+ (ref = HR−)	0.38	(0.34, 0.43)	<0.001
Histology (ref = ductal)			
Lobular	0.83	(0.67, 1.02)	0.072
Other	0.87	(0.60, 1.27)	0.467
Grade (ref = well-differentiated)			
2	1.59	(1.20, 2.11)	0.001
3–4	2.17	(1.64, 2.88)	<0.001
Age category (ref = 18–39)			
40–69	0.97	(0.83, 1.14)	0.717
70+	0.73	(0.58, 0.93)	0.011
Race (ref = White)			
Black	0.97	(0.81, 1.15)	0.724
Other	1.37	(1.10, 1.71)	0.005
Year of diagnosis (ref = 2013)			
2014	1.19	(1.02, 1.40)	0.032
2015	1.25	(1.07, 1.45)	0.005
Charlson Comorbidity Index (ref = 0)			
1	0.82	(0.68, 0.99)	0.036
2	0.77	(0.50, 1.17)	0.222
≥3	0.80	(0.42, 1.55)	0.515

*pCR* pathologic complete response, *NAC* neoadjuvant chemotherapy.

^a^All adjusted variables in the analysis are included in the table.

### Factors associated with mortality stratified by response to chemotherapy

Using 3-year overall survival data, in bivariate analysis, pCR was significantly associated with mortality, with 3-year overall survival in patients with pCR of 97.7% versus 94.4% in patients with residual disease (log-rank *p* < 0.001). On multivariable analysis prior to stratification by response to chemotherapy, pCR was significantly associated with improved survival (HR 0.25, 95% CI 0.16–0.41, *p* < 0.001). To determine factors, such as clinical T stage and type of chemotherapy, associated with survival after accounting for response to chemotherapy, we created separate models for patients that achieved a pCR and those with residual disease to examine independence of these factors from response to chemotherapy. In the multivariable Cox model (Table 4), in patients with pCR, higher comorbidity was associated with mortality, compared with those with no comorbidities (Charlson Comorbidity Index = 2: hazard ratio [HR] 10.87, 95% CI 2.23–52.96; Charlson Comorbidity Index ≥3, HR 8.46, 95% CI 1.02–70.31). Notably, no other factors, including clinical T stage, chemotherapy regimen, grade, or age were significantly associated with risk of mortality. We similarly analyzed only cT2 patients stratified by pCR or residual disease (Supplementary Table 2). Analysis of only cT2 patients did not differ significantly from the analysis including cT1 and cT2 patients.

**Table 4:** Cox proportional hazard models of predictors of mortality among patients with pCR and residual disease after NAC and HER2-targeted therapy^a^.

	pCR			Residual disease		
	HR	(95% CI)	*p*-value	HR	(95% CI)	*p*-value
cT2 stage (ref = cT1)	1.66	(0.55, 5.00)	0.371	0.80	(0.49, 1.31)	0.378
pT stage (ref = pT0/pTis)						
pT1	N/A	N/A	N/A	1.29	(0.29, 5.62)	0.739
pT2-4	N/A	N/A	N/A	3.21	(0.74, 14.04)	0.121
pN+ (ref = pN0)	N/A	N/A	N/A	3.41	(2.13, 5.47)	<0.001
Multi-agent NAC (ref = single-agent)	0.70	(0.21, 2.34)	0.558	2.51	(1.00, 6.35)	0.051
Hormone receptor+ (ref = HR−)	0.39	(0.15, 1.04)	0.060	0.43	(0.27, 0.70)	<0.001
Grade (ref = 1)						
2	0.32	(0.04, 2.76)	0.298	0.92	(0.32, 2.64)	0.878
3–4	0.36	(0.04, 2.90)	0.334	1.19	(0.42, 3.36)	0.739
Lobular histology (ref = ductal)	0.83	(0.11, 6.57)	0.862	0.79	(0.35, 1.75)	0.558
Age category (ref = 18–39)						
40–69	0.52	(0.14, 1.92)	0.324	0.78	(0.42, 1.46)	0.443
70+	2.67	(0.61, 11.64)	0.192	2.93	(1.40, 6.12)	0.004
Black Race (ref = White)	1.66	(0.57, 4.84)	0.351	1.13	(0.55, 2.31)	0.738
Year of diagnosis (ref = 2013)						
2014	1.04	(0.36, 2.98)	0.942	0.87	(0.51, 1.48)	0.600
2015	0.71	(0.17, 3.02)	0.645	0.74	(0.37, 1.47)	0.390
Charlson Comorbidity Index (ref = 0)						
1	2.57	(0.78, 8.47)	0.122	1.14	(0.58, 2.24)	0.705
2	10.87	(2.23, 52.96)	0.003	1.38	(0.42, 4.52)	0.593
≥3	8.46	(1.02, 70.31)	0.048	6.64	(1.51, 29.09)	0.012

*pCR* pathologic complete response, *NAC* neoadjuvant chemotherapy.

^a^All adjusted variables in the analysis are included in the table.

**Fig. 1:**
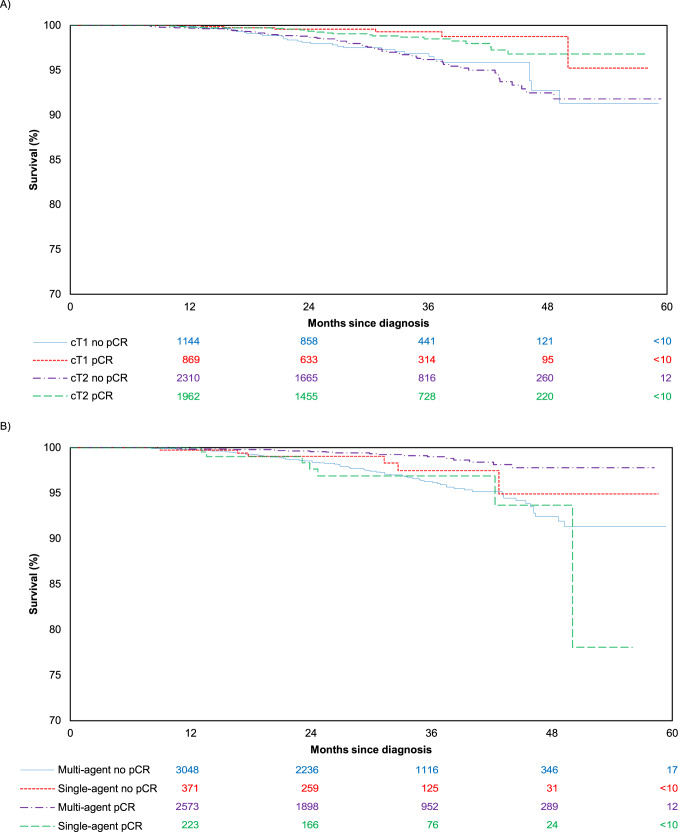
Overall survival in patients with cT1-T2 N0 HER2-amplified breast cancer treated with neoadjuvant chemotherapy. **A** cT stage was not associated with survival in patients that achieved a pCR or those with residual disease, and **B** single or multi-agent chemotherapy was not associated with survival in patients that achieved a pCR or those with residual disease.

In the multivariable Cox model in patients with residual disease after NAC, positive node (HR 3.41, 95% CI 2.13–5.47), age >70 in comparison to age 18–39 (HR 2.93, 95% CI 1.40–6.12), and Charlson Comorbidity Index ≥3 in comparison to 0 (HR 6.64, 95% CI 1.51–29.09) were associated with mortality, while hormone receptor positivity was associated with improved survival (HR 0.43, 95% CI 0.27–0.70; Table 4). Again, of note, clinical T stage was not associated with survival. Multi-agent chemotherapy, compared with single-agent, was associated with worse survival among women with residual disease, but estimates were imprecise (HR 2.51, 95% CI 1.00–6.35). The Kaplan–Meier analysis of survival, stratified by pCR versus residual disease and clinical T stage, is shown in Fig. 1A. The survival analysis stratified by pCR and type of chemotherapy (single versus multi-agent) chemotherapy is shown in Fig. 1B.

## Discussion

The ideal strategy to select patients safe for de-escalation of chemotherapy while identifying patients in need of more aggressive regimens in early node-negative HER2-amplified breast cancer is not clear. To inform this question, we report in a large observational cohort the relationship of single versus multi-agent chemotherapy to pathologic complete response and factors associated with mortality in patients that achieved pCR and those with residual disease.

In patients with clinically small, node-negative breast cancers, pathologic complete response was achieved more often with multi-agent chemotherapy compared to single agent. We report overall pCR rate of 37% in patients treated with single-agent chemotherapy with HER2-directed therapy, which is in the high range of rates reported in the TH arms of prospective trials examining more advanced disease, including NeoSphere (21.5%), NeoALTTO (27%), and CALBG 40601 (45%)^22–24^. All three trials included arms with dual HER2-directed therapy (addition of either lapatinib or pertuzumab), which significantly increased the pCR rate over TH. Pertuzumab was approved by the FDA following our enrollment period, and the addition of a second HER2-directed agent may increase the pCR rates for early disease over those reported in our study.

Although overall survival data are immature, in this study pCR was associated with improved survival in patients with early-stage disease. The prognostic value of pCR has been well established in neoadjuvant trials of more advanced disease^25^, but these data indicate that pCR is a biologically informative prognostic marker in early disease as well, although the effect is small in this short timeframe. In patients that achieved a pCR, no cancer-related factors were associated with short term survival, which was only associated with comorbidities. In contrast, in patients with residual disease, tumor features including hormone receptor status and positive lymph nodes were predictive of survival, indicating that cancer mortality may be a driver of overall mortality in these patients even in this short time period. Similar to more advanced disease, these data indicate that residual disease after neoadjuvant chemotherapy in patients with early HER2+ breast cancer identifies patients at increased risk of death. Clinical T stage, which is commonly used to determine treatment (multi-agent NAC versus surgery first) in N0 patients, was not prognostic in patients achieving pCR or those with residual disease, indicating this may not be the ideal selection criterion^26^. Response to chemotherapy is a biologically informative prognostic marker and could be a better stratification point to inform which patients need more or less aggressive systemic therapy.

Balancing potential benefits and side effects of chemotherapy is a key therapeutic decision. The toxicity of chemotherapy is an important component in decisions regarding continuation of therapy^27,28^. In this study, multi-agent chemotherapy was associated with an absolute increase in pCR rate of 9% compared to single agent with a modest odds ratio compared to hormone receptor status and grade. Depending on long-term survival difference between women with pCR versus residual disease and the ability to mitigate that difference with adjuvant therapy, increased rates of pCR in patients treated with multi-agent chemotherapy may not routinely justify increased toxicity upfront^29^. However, the relationship between pCR and survival is not uniform. Patients with hormone receptor-positive tumors are less likely to have a pCR but have improved survival. Using pCR to determine risk of recurrence may be less useful in ER+/HER2+ disease, especially in early disease where patients may have excellent outcomes with de-escalated therapy regardless of pCR status.

In patients with residual disease, having received upfront multi-agent chemotherapy was a predictor of poor survival. This may reflect selection of patients expected to have worse outcome for multi-agent regimens, that residual disease after multi-agent NAC may be biologically worse than residual disease after single-agent NAC, or related to adverse effects of multi-agent chemotherapy. Alternatively, patients that were treated more aggressively upfront may have fewer therapeutic options in the adjuvant setting.

We found that APT eligible patients with T1 tumors, with residual disease after NAC have similar survival to patients with T2 tumors with residual disease and may represent a higher risk group than was representative of that trial. Response to therapy may contribute to shared decision-making with patients regarding an adjuvant regimen that balances the risks of additional therapy with anticipated outcomes^15^. More study is needed to determine optimal escalation strategies, and patients that do not need additional adjuvant therapy, for APT eligible patients residual disease after neoadjuvant chemotherapy.

This study has several important limitations. NCBD data is incomplete. Information from only facilities with CoC accreditation is included, participation is voluntary, and data are reported by facilities without additional review. In addition, small sample sizes in the stratified analyses led to imprecise estimates. In this study, a minority of patients received single-agent chemotherapy, and the reasons for which those patients were selected for that therapy by the treating teams are not fully captured in the NCDB. Furthermore, information about specific therapeutic agents is missing and we are not able to determine adjuvant therapy. Lastly, recurrence data is not available from the NCDB. We report only 3-year overall survival, which may be insufficient for differences in outcomes to manifest.

In this large observational cohort of early HER2+ breast cancer, multi-agent chemotherapy was associated with higher likelihood of pCR, but this effect was modest compared to other factors. Single-agent NAC with HER2-directed therapy resulted in a pCR in a substantial number of patients and may provide excellent outcome with reduced toxicity, while allowing escalated therapy in the adjuvant setting for patients with residual disease. Prospective studies are needed to determine effects of de-escalation in the neoadjuvant setting, identify patients safe for de-escalation strategies, and determine optimal escalation strategies for patients with residual disease.

## Methods

### Data source

We utilized data from the American College of Surgeons and American Cancer Society 2016 National Cancer Database (NCDB) breast cancer participant use file. The NCDB is a clinical oncology database covering over 70% of incident cancers in the U.S.^18^. Data are collected from over 1500 Commission on Cancer (CoC) accredited facilities. We followed best practices for analyses using NCDB^19^.

### Study design and population

We identified adult women (≥18 years) with clinical T1N0 or T2N0 HER2+ malignant carcinoma of the breast between January 1, 2013 and December 31, 2015. Women were followed for up to 3 years after diagnosis. Prior years were not included due to changes in definitions for key study variables in 2013. Women were included if they were treated with NAC with HER2-targeted therapy prior to surgery and underwent either a mastectomy or breast-conserving therapy within 8 months of their breast cancer diagnosis. HER2-targeted therapy was identified using the NCDB immunotherapy variable. Women who died or were lost to follow-up within 8 months of diagnosis were excluded.

### Pathologic response

Pathologic response was categorized as pathologic complete response (pCR, ypT0N0/ypTisN0). Individuals with missing pathologic T or N stage were treated as missing.

### Covariates

Demographic and clinical covariates were assessed for analyses of factors associated with pCR and mortality. Demographic factors included age and race. Tumor factors included hormone receptor status, tumor grade, histology, and year of diagnosis. Comorbidity burden was assessed using the Charlson Comorbidity Index (CCI) in the NCDB file. The NCDB contains an adjuvant therapy data field, but there is only specific data on the first course of chemotherapy which in this cohort was neoadjuvant. It is additionally a composite variable of chemotherapy, hormone therapy, and immunotherapy, and was missing for the majority of patients in this study, so we did not include it in the analysis.

### Statistical analysis

Patient demographic and clinical characteristics were described using frequency statistics for categorical variables and medians and interquartile ranges (IQRs) for continuous variables. Pathologic response rates were described using frequency statistics, stratified by type of NAC (single vs. multi-agent). Chemotherapy is reported in the NCDB as single or multi-agent and we are unable to identify patients treated with specific drugs or regimens. HER2-directed therapy is reported separately, and so we can include only patients that received HER2-directed therapy and stratify those patients by single or multi-agent chemotherapy regimens. Factors associated with pCR to neoadjuvant therapy were assessed using a multivariable logistic regression model that included overall pCR as the dependent variable and patient demographic and clinical factors as independent variables.

Kaplan–Meier analyses were used to assess survival, stratifying by type of NAC (single vs. multi-agent) and pathologic response (pCR vs. no pCR). A landmark approach was used to address potential immortal time bias^20,21^. Follow-up for survival analyses was started for all women at 8 months following diagnosis, to account for variable timing of surgery and NAC initiation. To determine the significance of other clinical and pathologic features in addition to the prognostication provided by pCR, we analyzed factors associated with mortality using Cox proportional hazards regression models. Separate models were fit in women with pCR and those with residual disease. Individuals with missing covariate information were excluded from all models. All analyses were conducted using SAS Version 9.4 (SAS Inc., Cary, NC). The study was exempted from review by the Institutional Review Board at the University of North Carolina at Chapel Hill (IRB# 20-1493).

### Reporting summary

Further information on research design is available in the Nature Research Reporting Summary linked to this article.

## Supplementary information

Supplementary Files

Consort Checklist

Reporting Summary

NIH Publishing Agreement

## Data Availability

The National Cancer Database Participant User Files can be requested from the American College of Surgeons: https://www.facs.org/quality-programs/cancer/ncdb/puf.

## Code availability

Code used for analysis is available upon request.
