# Supplementary material for: Pathologic complete response and survival after neoadjuvant chemotherapy in cT1-T2/N0 HER2+ breast cancer
Source: NPJ Breast Cancer. 2022 May 12;8:65. doi: 10.1038/s41523-022-00433-x (PMC9098414; doi:10.1038/s41523-022-00433-x)
Supplement: Supplementary file 1 — Supplementary Files [file 41523_2022_433_MOESM1_ESM.pdf]

Supplemental Table. Response to NAC in cT1 patients stratified by T stage

	Single-agent NAC*		Multi-agent NAC*	
	cT1a/b n=69	cT1c n=170	cT1a/b n=328	cT1c n=1,397
pCR** (no, %)§	39 (60.9)	67 (45.3)	125 (42.5)	542 (43.3)

* NAC: neoadjuvant chemotherapy
** pCR: pathologic complete response (pT0 pN0)

Supplemental Table 2. Cox proportional hazard models in cT2 patients of predictors of mortality among patients with pCR* and residual disease after NAC** and HER2-targeted therapy†

	pCR			Residual Disease		
	HR	(95% CI)	p-value	HR	(95% CI)	p-value
pT stage (ref=pT0/pTis)						
pT1	N/A	N/A	N/A	1.99	(0.25, 15.65)	0.51
pT2-4	N/A	N/A	N/A	4.96	(0.64, 38.44)	0.12
pN+ (ref=pN0)	N/A	N/A	N/A	3.36	(1.93, 5.87)	<0.001
Multi-agent NAC** (ref=single-agent)	0.60	(0.20, 1.85)	0.38	4.00	(0.96, 16.78)	0.06
Hormone receptor + (ref=HR-)	0.38	(0.14, 1.01)	0.05	0.39	(0.22, 0.68)	<0.001
Lobular histology (ref=ductal)	0.76	(0.09, 6.17)	0.79	0.84	(0.35, 2.00)	0.69
Age category (ref=18-39)						
40-69	0.80	(0.17, 3.69)	0.77	0.79	(0.39, 1.60)	0.51
70+	5.49	(1.08, 27.84)	0.04	3.21	(1.37, 7.49)	0.01
Black Race (ref=White)	1.30	(0.41, 4.11)	0.66	1.41	(0.66, 3.05)	0.38
Year of diagnosis (ref=2013)						
2014	0.70	(0.23, 2.17)	0.54	0.65	(0.35, 1.22)	0.18
2015	0.99	(0.27, 3.57)	0.98	0.81	(0.37, 1.78)	0.59
CCI (ref=0)						
1	1.92	(0.59, 6.27)	0.28			
2	8.38	(1.80, 39.05)	0.01	0.89	(0.37, 2.10)	0.78
≥3	7.86	(0.94, 65.48)	0.06	1.64	(0.49, 5.49)	0.42

* pCR: pathologic complete response
** NAC: neoadjuvant chemotherapy
† All adjusted variables in the analysis are included in the table.
CCI: Charlson Comorbidity Index
